# Particulate matter facilitates amphiregulin-dependent lung cancer proliferation through glutamine metabolism

**DOI:** 10.7150/ijbs.96210

**Published:** 2024-05-27

**Authors:** Ya-Jing Jiang, Trung-Loc Ho, Chia-Chia Chao, Xiu-Yuan He, Po-Chun Chen, Fang-Ju Cheng, Wei-Chien Huang, Chang-Lun Huang, Po-I Liu, Chih-Hsin Tang

**Affiliations:** 1 Graduate Institute of Biomedical Sciences, China Medical University, Taichung, Taiwan.; 2 Department of Respiratory Therapy, Fu-Jen Catholic University, New Taipei City, Taiwan.; 3 Department of Life Science, National Taiwan Normal University, Taipei, Taiwan.; 4 Center for Molecular Medicine, China Medical University Hospital, Taichung, Taiwan.; 5 Department of Medical Research, China Medical University Hsinchu Hospital, Hsinchu, Taiwan.; 6 Department of Medical Laboratory Science and Biotechnology, Asia University, Taichung, Taiwan.; 7 Division of General Thoracic Surgery, Department of Surgery, Changhua Christian Hospital, Changhua, Taiwan.; 8 Department of Physical Therapy, Asia University, Taichung, Taiwan.; 9 Department of General Thoracic Surgery, Asia University Hospital, Taichung, Taiwan.; 10 Department of Pharmacology, School of Medicine, China Medical University, Taichung, Taiwan.; 11 Chinese Medicine Research Center, China Medical University, Taichung, Taiwan.

**Keywords:** Lung cancer, Particulate matter, Glutamine metabolism, Amphiregulin, SLC1A5

## Abstract

Although many cohort studies have reported that long-term exposure to particulate matter (PM) causes lung cancer, the molecular mechanisms underlying the PM-induced increases in lung cancer progression remain unclear. We applied the lung cancer cell line A549 (Parental; A549.Par) to PM for an extended period to establish a mimic PM-exposed lung cancer cell line, A549.PM. Our results indicate that A549.PM exhibits higher cell growth and proliferation abilities compared to A549.Par cells *in vitro* and *in vivo*. The RNA sequencing analysis found amphiregulin (AREG) plays a critical role in PM-induced cell proliferation. We observed that PM increases AREG-dependent lung cancer proliferation through glutamine metabolism. In addition, the EGFR/PI3K/AKT/mTOR signaling pathway is involved in PM-induced solute carrier family A1 member 5 (SLC1A5) expression and glutamine metabolism. Our findings offer important insights into how lung cancer proliferation develops upon exposure to PM.

## 1. Introduction

Air pollution is a global issue that affects people worldwide. In recent years, the impact of air pollution on health has garnered increasing attention 1. The World Health Organization (WHO) estimated that the combined impacts of indoor and outdoor air pollution result in approximately 7 million premature deaths annually 2. Particulate matter (PM) is a key indicator of air pollution introduced into the air by various natural and human activities 3. Research conducted in many regions of the world indicates that air pollution is associated with an increasing number of adverse health effects. Exposure to PM also has been identified as the cause of numerous health effects including respiratory symptoms, exacerbation of chronic respiratory and cardiovascular diseases 4, 5. The International Agency for Research on Cancer (IARC), in a meta-analysis of findings from 14 studies of outdoor air pollution conducted largely in North America and Europe, reported a statistically significant 9% increase in risk for lung cancer incidence or mortality per each 10 µg/m^3^ increase in PM2.5 concentrations 6. PM also induces cell migration, invasion, and epithelial-mesenchymal transition (EMT) in lung cancer cells, thereby promoting tumor metastasis 7. Although the relationship between PM and lung cancer is established, the underlying mechanism remains unclear.

Long-term exposure to PM2.5 causes DNA damage and activates AhR, epidermal growth factor receptor (EGFR), and the immune system, leading to tumorigenesis 8. Amphiregulin (AREG), is an epidermal growth factor ligand of EGFR 9, further promoting distant metastasis, anti-apoptosis, and drug resistance in cancer 10. AREG has been found to play a role in various physiological processes, including breast development, bone formation, cell invasion, and angiogenesis 11-13. AREG stimulates cell migration and proliferation while reducing lung cancer cell apoptosis 14. Moreover, AREG is linked to a worse prognosis and shorter survival in lung cancer patients 15. Therefore, AREG plays a crucial role in regulating lung cancer cell growth, differentiation, and metastasis.

Previous studies have indicated that tumor cells adapt to the tumor microenvironment by altering their metabolic pathways 16. Tumor cells mainly obtain nutrients through metabolic pathways involving glucose, fatty acids, glutamine, and small-molecule amino acids to support tumor cell growth 17. Glutamine is considered central to cell growth and metabolism in both normal and cancer cells. Moreover, glutamine participates in cell signaling pathways to promote hallmarks of malignancies, including sustaining proliferation, invasion, and metastasis 18. Solute carrier family A1 member 5 (SLC1A5) is the primary transporter of glutamine 19. The SLC1A5 variation is essential for the metabolic reprogramming of cancer because it transports glutamine into mitochondria 20. In the glutamine metabolic pathway, glutamine enters tumor cells through SLC1A5, where it is converted into glutamate by glutaminase and then enters the mitochondria. Glutamate dehydrogenase converts glutamate in the mitochondria into α-ketoglutarate (α-KG), which enters the tricarboxylic acid (TCA) cycle to provide energy to tumor cells and promote tumor cell growth 21-24. However, the association of metabolism with PM and lung cancer has not been widely explored.

Our research sought to determine whether long-term exposure to PM through metabolic pathways promotes lung cancer cell proliferation. We applied the lung cancer cell line A549 (Parental; A549.Par) to PM (25 μg/ml) for an extended period to establish a mimic PM-exposed lung cancer cell line (A549.PM). Our *in vitro* and *in vivo* evidence showed that long-term exposure to PM promotes lung cancer growth. PM upregulates AREG expression through the EGFR/PI3K/AKT/mTOR signaling pathway, leading to an increase in the expression of the glutamine transporter SLC1A5. This promotion of SLC1A5 enhances mitochondrial respiration via the glutamine-derived TCA cycle metabolites, consequently stimulating the proliferation of lung cancer cells.

## 2. Materials and Methods

The detailed sources of antibodies, chemicals, cell culture, quantitative real-time polymerase chain reaction (RT-qPCR), western blot, ELISA, Seahorse and immunohistochemistry (IHC) staining assays are all obtainable within Supplementary Materials and Methods.

### 2.1 PM

The PMs were standard reference material 1649b (SRM1649b) purchased from the National Institute of Standards and Technology NIST (MD, USA). The PMs consisted of urban dust. They were dispersed in ultra-pure water and sonicated for 30 minutes in an ultrasonic bath before being used. All PMs were stored at 4 °C.

### 2.2 Cell viability assay

Cell proliferation was determined by the 3-(4,5-dimethylthiazol-2-yl)-2,5-diphenyltetrazolium bromide (MTT) assay and cell counting kit-8 (CCK-8) assay (ab228554, Abcam, Burlingame, CA, USA). Cells were transfected with AREG or SLC1A5 shRNA at 24 hr. In the MTT assay, cells were seeded in 96 well plates at a concentration of 2000 cells per well for 24 hr and 48 hr. After cultures were washed with PBS, 0.5 mg/ml of MTT solution was added and incubated for 1 hr at 37°C. The formazan crystals were dissolved in 50 μl of DMSO and the absorbance was measured at 450 nm 25, 26. In the CCK-8 assay, cells were seeded in 96 well plates at a concentration of 3000 cells per well for 24 hr and 48 hr. After cultures, the CCK8 regent was added and incubated for 2 hr at 37 °C, the absorbance was measured at 450 nm.

### 2.3 Colony formation assay

Cells were transfected with AREG or SLC1A5 shRNA. After 24 hr, A549.PM cells were re-plated in six-well plates at a density of 3000 cells per well and cultured with DMEM supplemented with 10% FBS for 7 days. At the end of the incubation period, the cells were washed twice with PBS, fixed in methanol, stained with 5% crystal violet, and manually counted under a microscope 27.

### 2.4 RNA Sequencing (RNA-Seq) and data analysis

Total RNA of the A549.Par and A549.PM cells were isolated for RNA sequencing. RNA quality and integrity were examined using Bioanalyzer 2100 and RNA 1000 Nano LabChip Kit (Agilent); samples with an RNA integrity number less than 7 were excluded from the subsequent assay. After the mRNA fragmented and cDNA library preparation, RNA sequencing was conducted using the Illumina HiSeq 4000 (paired-end, 150 base pairs, PE150) and mapped by using the HISAT package (http://ccb.jhu.edu/software/hisat2). EdgeR was utilized to estimate the differentially regulated genes of all transcripts by calculating fragments per kilobase per million (FPKM). Differentially expressed genes were determined with a log2 (fold change) >1 or log2 (fold change) <-1 and statistical significance (*p*-value < 0.05) using the R package 28.

### 2.5 Meta-analysis of microarray datasets from The Cancer Genome Atlas (TCGA) database

Using the TCGA dataset of lung adenocarcinoma cancers, we identified 48 patients analyzed for AREG gene expression and 57 patients analyzed for SLC1A5 gene expression for each tumor sample.

### 2.6 Measurement of Oxygen Consumption Rate (OCR)

A549.Par and A549.PM cells (2 × 10^4^ cells/well) were seeded in Seahorse XF24-well microplate (Agilent). After the seeding period, cells were cultured in an assay medium without sodium bicarbonate and HEPES in a non-CO_2_ incubator at 37 °C for 1 hr. OCR was examined using Seahorse XF Cell Mito Stress Test Kit (103015-100, Agilent, Santa Clara, CA, USA). After baseline measurements, for OCR, oligomycin, the reversible inhibitor of oxidative phosphorylation FCCP (p-trifluoromethoxy carbonyl cyanide phenylhydrazone), and the mitochondrial complex I inhibitor rotenone plus the mitochondrial complex III inhibitor antimycin A (Rote/AA) were sequentially injected. Data were assessed by Seahorse XF24 Analyzer (Agilent, Santa Clara, CA, USA) 29.

### 2.7 Glutamine levels

The concentration of glutamine was measured using a Glutamine Detection Assay Kit (Abcam, Burlingame, CA, USA) following the manufacturer's instructions after culturing cells (1 × 10^4^/well) in 24-well plates 29.

### 2.8 Mass spectrometry by carbon-13 tracing experiments

A549.Par or A549.PM cells are cultured for 24 hours in glucose/glutamine-free Dulbecco MEM which is either fully labeled 13C-glutamine or 1,6-13C glucose. After 24 hours, remove the media, and wash the cells with PBS twice then detect the relative amounts of metabolites (from glycolysis or glutamine metabolism) in the cells.

### 2.9 *In vivo* tumor growth assay

Mice will be subcutaneously injected with 0.2 mL Matrigel containing 5 × 10^6^ A549. Par or A549.PM cells. After injection, the tumor development was monitored by IVIS imaging system (Xenogen, UK) every week. After 4 weeks, the mice were sacrificed by CO_2_ inhalation and the tumors were removed, fixed with 4% paraformaldehyde/PBS, and embedded in paraffin. Subsequently, the samples will be processed for AREG and SLC1A5 staining.

### 2.10 Statistical analysis

All values are expressed as the mean ± standard deviation (SD). Statistical differences between the experimental groups were assessed for significance using the Student's t-test. Statistical comparisons of more than two groups were performed using one-way analysis of variance (ANOVA) with Bonferroni's post hoc test. Between-group differences were significant if the *p*-value was less than 0.05.

## 3. Results

### 3.1 PM-induced lung cancer progression *in vitro* and *in vivo*

**Figure 1:**
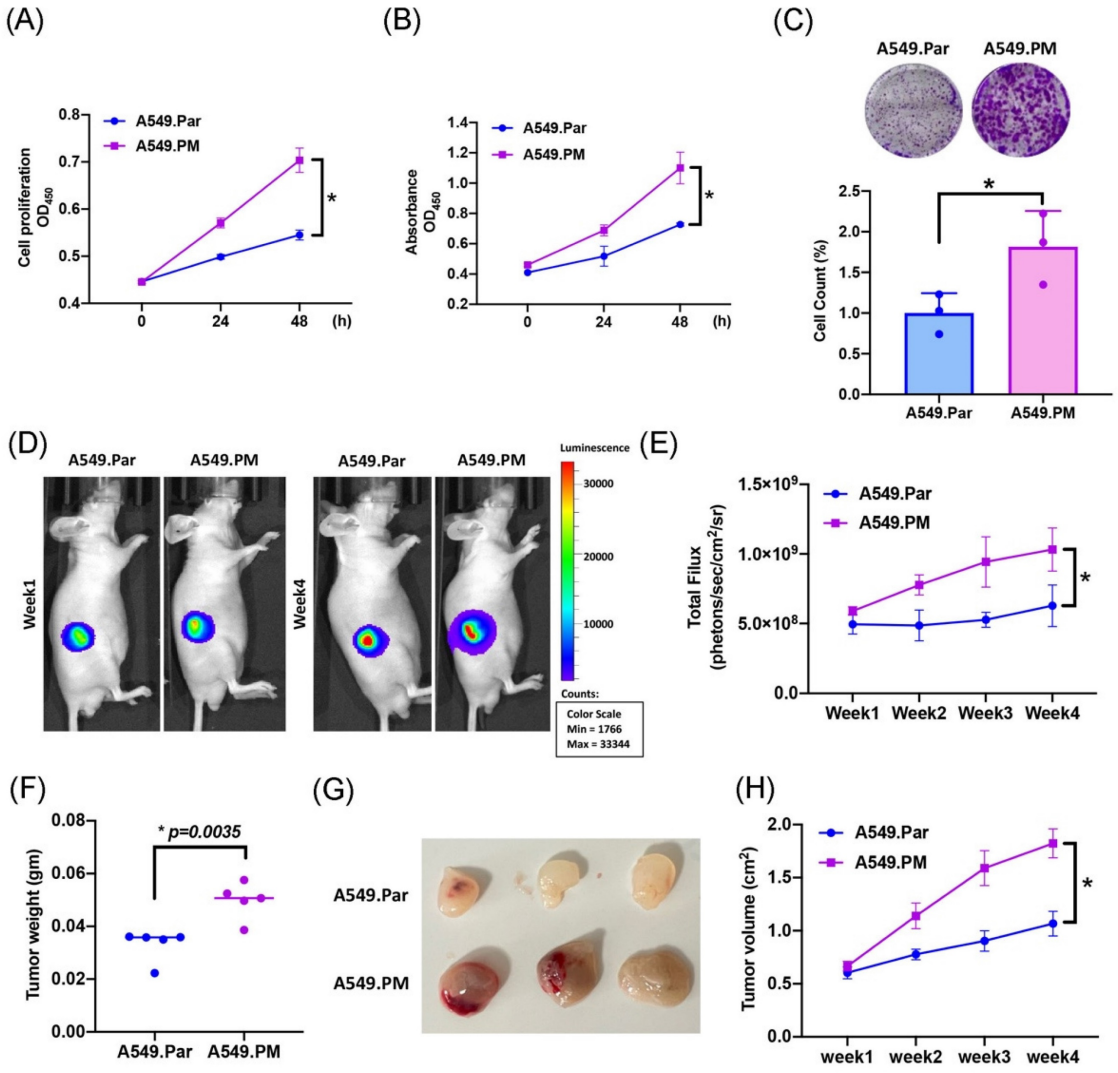
** PM increases cell proliferation in lung cancer.** (A&B) The cell proliferation ability of A549.Par and A549.PM cells was analyzed by MTT and CCK-8 assays. (C) The cell growth of A549.Par and A549.PM cells was measured by colony assay. (D-F) A549.Par and A549.PM cells were subcutaneously injected into the right flanks of BALB/c-nu mice. Four weeks later, the mice were sacrificed, and the tumors were excised and weighed. (G&H) At week 4, the mice were sacrificed, and the tumors were excised and photographed. *p < 0.05 compared with A549.Par expression.

A previous study has shown that PM plays an important role in lung cancer proliferation and metastasis 30. To validate whether PM induced lung cancer proliferation, we exposed A549 cells (A549.Par) to 25 μg/mL PM for long-term exposure (A549.PM) to mimic PM exposure behavior. The result of the MTT and CCK-8 assay showed that A549.PM cells have a higher cell proliferation ability than A549.Par cells (Figure 1A&B). The growth rate was significantly enhanced in A549.PM cells compared with A549.Par cells, as demonstrated by the colony assay (Figure 1C). In our *in vivo* data, we found that A549.PM cells exhibit higher tumor growth than A549.Par cells (Figure 1D&E). Furthermore, A549.PM cells also increase tumor weight (Figure 1F) and tumor volume (Figure 1G&H).

### 3.2 Long-term exposure to PM increases AREG expression in lung cancer

**Figure 2:**
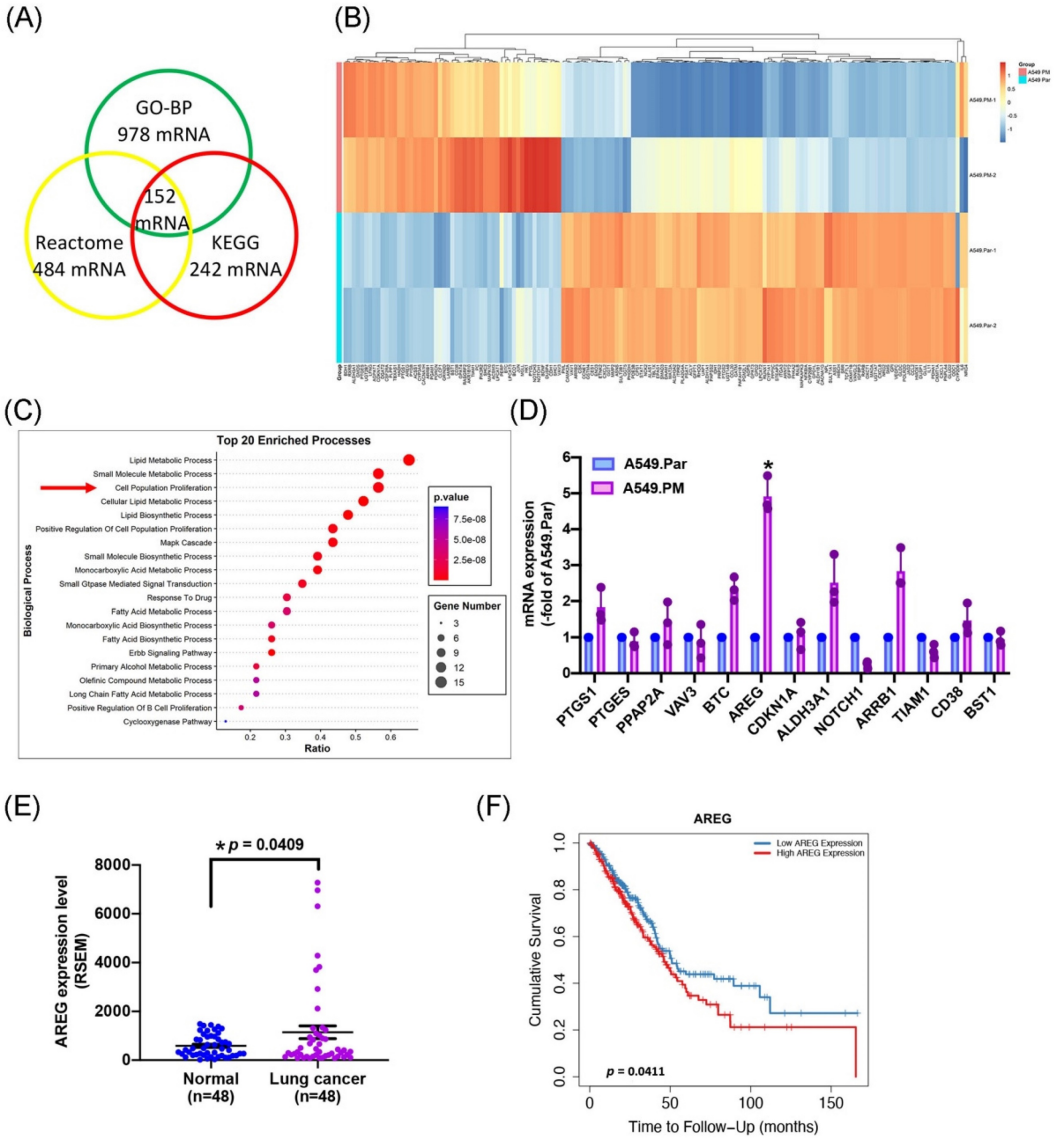
** PM promotes AREG expression in lung cancer cells.** (A&B) Overlap of genes in GO-BP, Reactome, and KEGG pathways expressed in the A549.PM cell line. The cluster heatmap shows the expression of 152 genes in the A549.PM cell line compared to the A549.Par cell line. (C) Gene ontology (GO) enrichment analysis of genes from the two groups. (D) The expression levels of selected up-regulated genes were verified by qPCR. (E) AREG mRNA expression in tumor tissue and adjacent normal tissue was analyzed using records from the TCGA database. (F) Timer 2.0 analysis determined the overall survival rates of patients with lung adenocarcinoma and levels of AREG expression. *p < 0.05 compared with A549.Par expression.

To identify the molecules responsible for the proliferative biological effects of PM, we performed RNA-Seq analysis in A549.Par and A549.PM cells. We found that Gene Ontology Biological Processes (GO-BP) (false discovery rate [FDR] < 0.01, fold change [FC] > 2) altered the expression of 978 genes, Reactome altered 484 genes, and KEGG altered 242 genes, with 152 genes altered under all three conditions (Figure 2A&B). RNA-Seq data also provided strong support for our earlier observations that PM promotes lung cancer cell proliferation (Figure 2C). These data confirm that long-term exposure to PM increases lung cancer cell growth and proliferation.

To determine which gene has the highest expression as predicted from the RNA-seq analysis in the proliferation group, the expression of mRNA was verified by qPCR. The results showed that PTGS1, BTC, AREG, ALDH3A1, and ARRB1 were secreted by A549.PM cells were upregulated compared to A549.Par cells. Among these genes, AREG levels in A549.PM cells were higher than all other up-regulated genes (Figure 2D). To examine the effects of AREG levels on lung cancer progression, we investigated the clinical significance of AREG identified in lung adenocarcinoma samples from the TCGA database. We found higher levels of AREG mRNA expression in tumor tissue than in adjacent normal tissue (Figure 2E). In Timer 2.0 analysis, progressively higher AREG expression among lung cancer patients was associated with correspondingly lower overall survival rates (Figure 2F). These results indicate that PM increases AREG expression in lung cancer cells.

### 3.3 PM-induced increases in AREG expression and facilitates lung cancer proliferation

**Figure 3:**
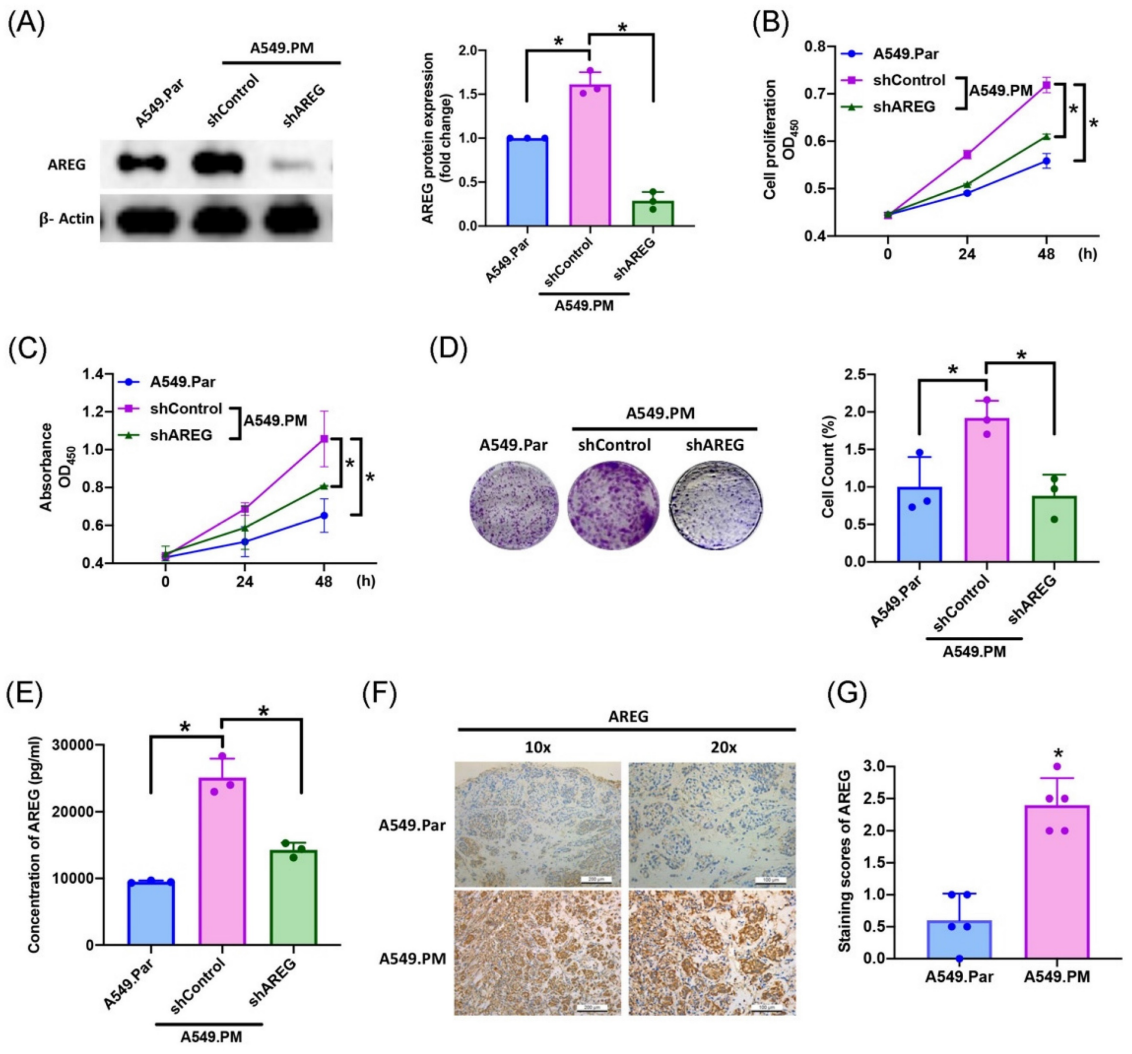
** PM-induced AREG expression enhances lung cancer growth and proliferation.** A549.PM cells were transfected with AREG shRNA for 24 hr. (A) Western blot analysis examined levels of AREG and β-actin. The graphs densitometric analysis of protein expression normalized to β-actin. (B&C) Cell proliferation was analyzed by MTT and CCK-8 assays for 2 days. (D) Cell growth ability was measured by colony assay. (E) Levels of AREG expression were examined by ELISA assays. (F&G) Tumors from sacrificed mice were stained with AREG and scored by intensity from 0 to 3 (where 0 = negative; 1 = weak; 2 = moderate; 3 = strong). *p < 0.05 compared with A549.Par or A549.PM expression.

In several cancers (ovarian, breast, and lung cancers), high AREG expression correlates with a worse prognosis 31-33. To validate whether the amount of AREG secreted from lung cancer cells affects cell proliferation, we found that long-term exposure to PM promotes lung cancer AREG protein expression, which is significantly decreased when transfected with AREG shRNA, as determined by western blot assay (Figure 3A). We also observed a significant decrease in cell proliferation ability as assessed by MTT and CCK-8 assays (Figure 3B&C). Colony assay results also showed that transfection with AREG shRNA significantly decreased cell growth ability (Figure 3D). According to ELISA results, PM exposure increased AREG expression, while transfection with AREG shRNA inhibited this phenomenon (Figure 3E). IHC staining of tumor tissue from *in vivo* study demonstrated that the expression of AREG significantly increased in the A549.PM group compared to the A549.Par group (Figure 3F&G). These results reveal that PM promotes AREG-dependent lung cancer proliferation.

### 3.4 PM-induced increases in AREG-dependent lung cancer proliferation through glutamine metabolism

**Figure 4:**
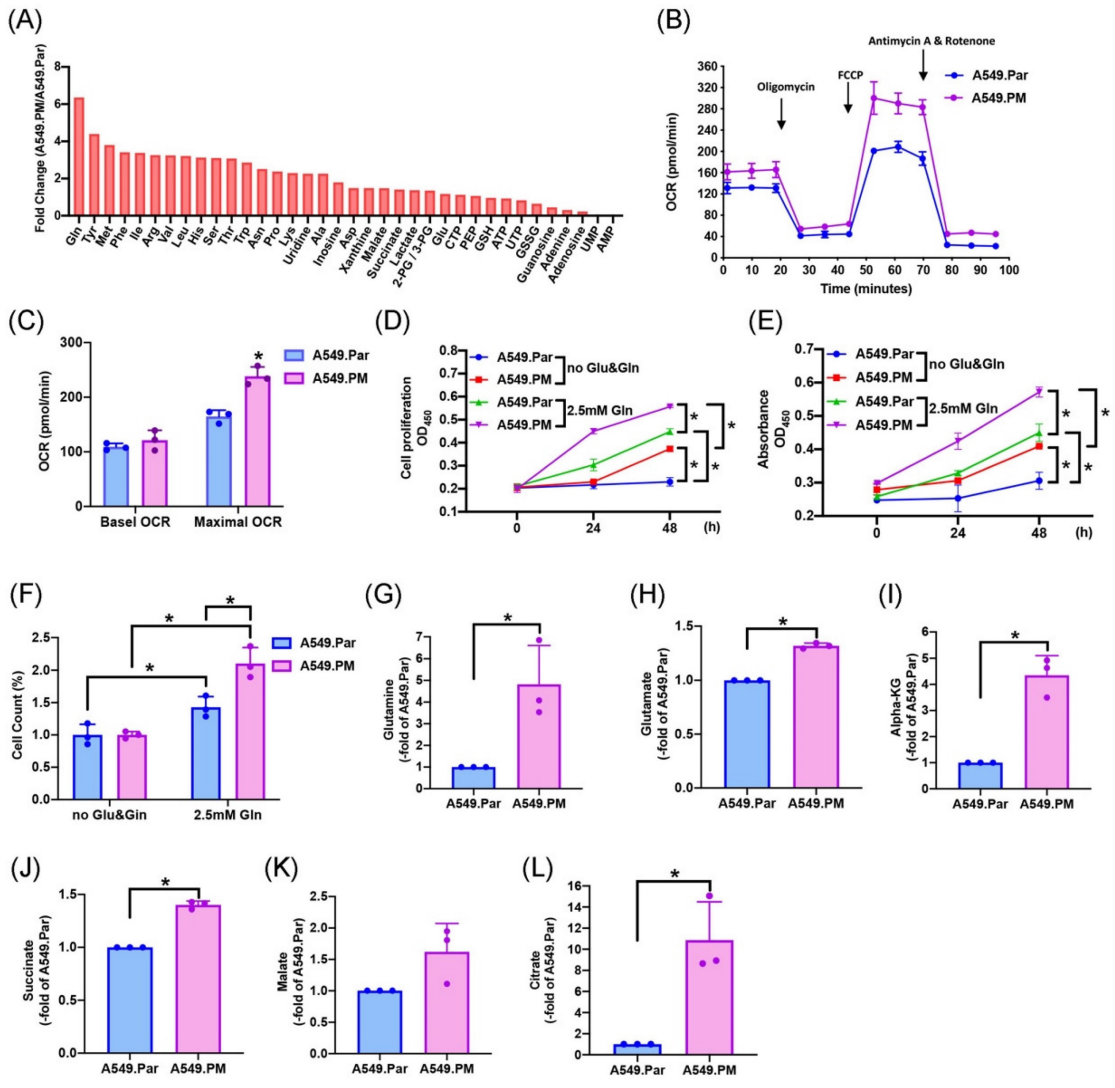
** PM increases TCA cycle metabolites through mitochondrial glutaminolysis, thereby increasing lung cancer glutamine metabolism and promoting cell proliferation.** (A) Metabolite concentration analysis in the mitochondria from A549.Par and A549.PM cells by mass spectrometry assay. (B&C) Oxygen consumption rate (OCR) was measured in A549.Par and A549.PM cells following the addition of oligomycin (1 µM), FCCP (0.5 µM), and the electron transport inhibitor rotenone/antimycin A (0.5 µM). (D&E) A549.Par and A549.PM cells were incubated with 2.5 mM glutamine. Cell proliferation was analyzed by MTT and CCK-8 assays for 2 days. (F) A549.Par and A549.PM cells were incubated with 2.5 mM glutamine for 7 days. Cell growth ability was measured by colony assay. (G-L) The metabolic abundance of glutamine-derived TCA metabolites (glutamine, glutamate, α-KG, succinate, malate, and citrate) in the purified mitochondria from A549.PM cells. *p < 0.05 compared with A549.Par or A549.PM expression.

**Figure 5:**
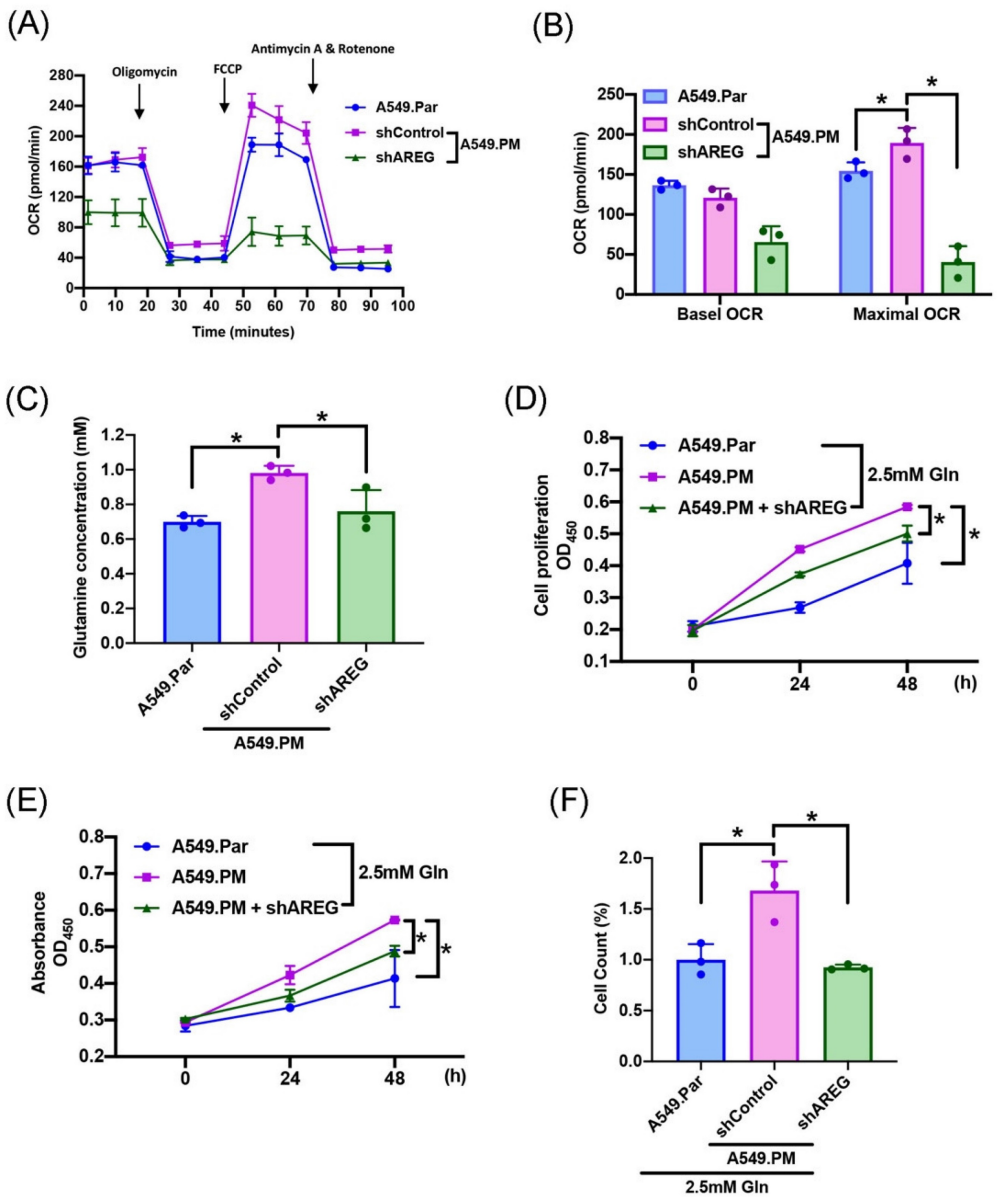
** PM stimulates the expression of AREG and facilitates the glutamine metabolism of lung cancer cells to encourage cell proliferation.** A549.PM cells were transfected with AREG shRNA for 24 hr. (A&B) Oxygen consumption rate (OCR) was measured by following the addition of oligomycin (1 µM), FCCP (0.5 µM), and electron transport inhibitor, rotenone/antimycin A (0.5 µM). (C) The concentration of glutamine in A549.Par and A549.PM cells. (D&E) A549.Par and A549.PM cells were incubated with 2.5 mM glutamine. The cell proliferation was analyzed by MTT and CCK-8 assays for 2 days. (F) A549.Par and A549.PM cells were incubated with 2.5 mM glutamine for 7 days. The cell growth ability was measured by colony assay. *p < 0.05 compared with A549.Par or A549.PM expression.

The two main sources of energy for cell development are the metabolism of glucose and glutamine 17. We sought to determine which metabolic pathway enhances lung cancer progression. Mass spectrometry data showed that glutamine concentration is higher in A549.PM cells than in A549.Par cells (Figure 4A). A549.PM cells also exhibited increased OCR compared to A549.Par cells (Figure 4B&C). In the MTT, CCK-8, and colony assay, cells were seeded in glucose and glutamine-free medium. We found that treating with 2.5 mM glutamine in both A549.Par and A549.PM cells resulted in higher cell growth and proliferation than in the glucose and glutamine-free groups (Figure 4D-F). Additionally, we found that A549.PM cells exhibit increased levels of glutamine-derived TCA cycle metabolites such as glutamine, glutamate, alpha-KG, succinate, malate, and citrate compared to the A549.Par group (Figure 4G-L). Our Seahorse and glutamine concentration assay showed that when A549.PM cells were transfected with AREG shRNA, there was a significant decrease in OCR and glutamine concentration (Figure 5A-C). We also found that when A549.PM cells were transfected with AREG shRNA, there was a significant decrease in cell growth and proliferation ability (Figure 5D-F). This finding implies that PM stimulates AREG expression and glutamine metabolism in lung cancer to encourage tumor cell growth.

### 3.5 PM increases AREG-dependent glutamate metabolism and lung cancer proliferation through SLC1A5

**Figure 6:**
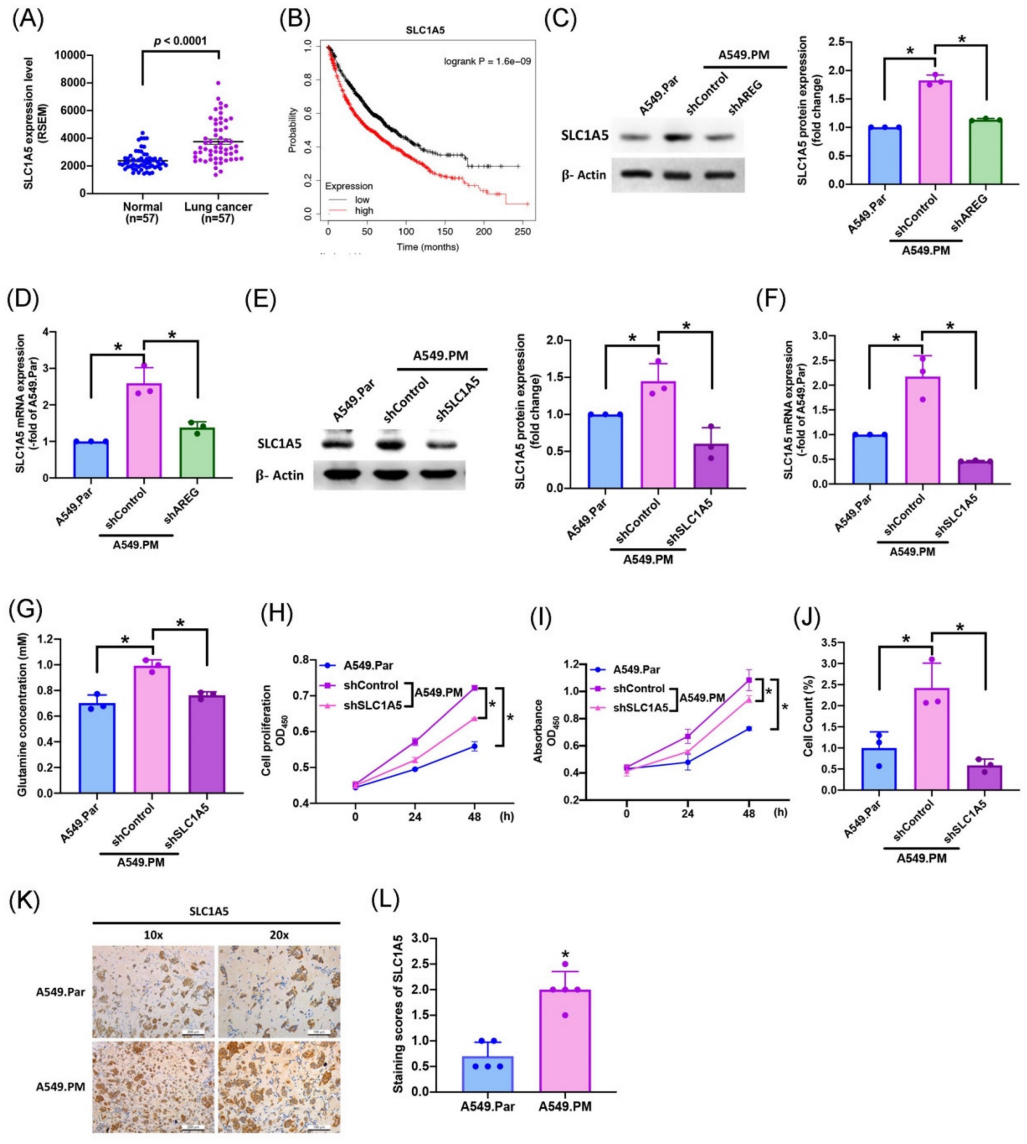
** PM increases AREG-dependent glutamate metabolism and lung cancer proliferation through SLC1A5.** (A) SLC1A5 mRNA expression in tumor tissue and adjacent normal tissue was analyzed using records from The Cancer Genome Atlas (TCGA) database. (B) Kaplan-Meier analysis determined levels of SLC1A5 expression and overall survival rates of patients with lung cancer. (C&D) A549.PM cells were transfected with AREG shRNA for 24 hr. The levels of SLC1A5 and β-actin were examined by Western blot analysis and the graphs densitometric analysis of protein expression normalized to β-actin, while the expression of SLC1A5 was verified using RT-qPCR. (E&F) A549.PM cells were transfected with SLC1A5 shRNA for 24 hr. The levels of SLC1A5 and β-actin were examined by Western blot analysis and the graphs densitometric analysis of protein expression normalized to β-actin, while the expression of SLC1A5 was verified using quantitative RT-qPCR. (G) The concentration of glutamine in A549.Par and A549.PM cells. (H&I) Cell proliferation was analyzed by MTT and CCK-8 assays for 2 days. (J) Cell growth ability was measured by colony assay. (K&L) Tumors from sacrificed mice were stained with SLC1A5 and scored by intensity from 0 to 3 (where 0 = negative; 1 = weak; 2 = moderate; 3 = strong). *p < 0.05 compared with A549.Par or A549.PM expression.

Several previous studies have shown that SLC1A5 is the most important transporter in glutamine metabolism 34, 35. To identify whether long-term PM exposure is associated with SLC1A5 levels in A549.PM cell lines, our data showed that levels of SLC1A5 mRNA expression were much higher in tumor tissue than in adjacent normal tissue (Figure 6A). In Kaplan-Meier analysis, progressively higher SLC1A5 expression among lung cancer patients was associated with correspondingly lower overall survival rates (Figure 6B). We found that when A549.PM cells were transfected with AREG shRNA, there was a significant decrease in SLC1A5 protein level and mRNA expression (Figure 6C&D). Additionally, long-term exposure to PM increased lung cancer SLC1A5 protein and mRNA expressions, which were significantly decreased when transfected with SLC1A5 shRNA, as determined by western blot and RT-qPCR assay (Figure 6E&F). Our glutamine concentration assay also showed that when A549.PM cells were transfected with AREG shRNA, there was a significant decrease in glutamine concentration (Figure 6G). Next, we aimed to examine whether the level of SLC1A5 promotes long-term PM exposure to lung cancer cell progression. We found that when transfected with SLC1A5 shRNA, there was a significant decrease in cell proliferation and growth by MTT, CCK-8 and colony assays compared with the A549.PM group (Figure 6H-J). *In vivo*, IHC staining of tumor tissue also demonstrated that the expression of SLC1A5 significantly increased in the A549.PM group compared to the A549.Par group (Figure 6K&L). These results reveal that PM-induced AREG expression through SLC1A5 affects glutamate metabolism and proliferation in lung cancer cells.

### 3.6 PM facilitates AREG-dependent glutamine metabolism through EGFR/ PI3K/AKT/mTOR signaling pathway

**Figure 7:**
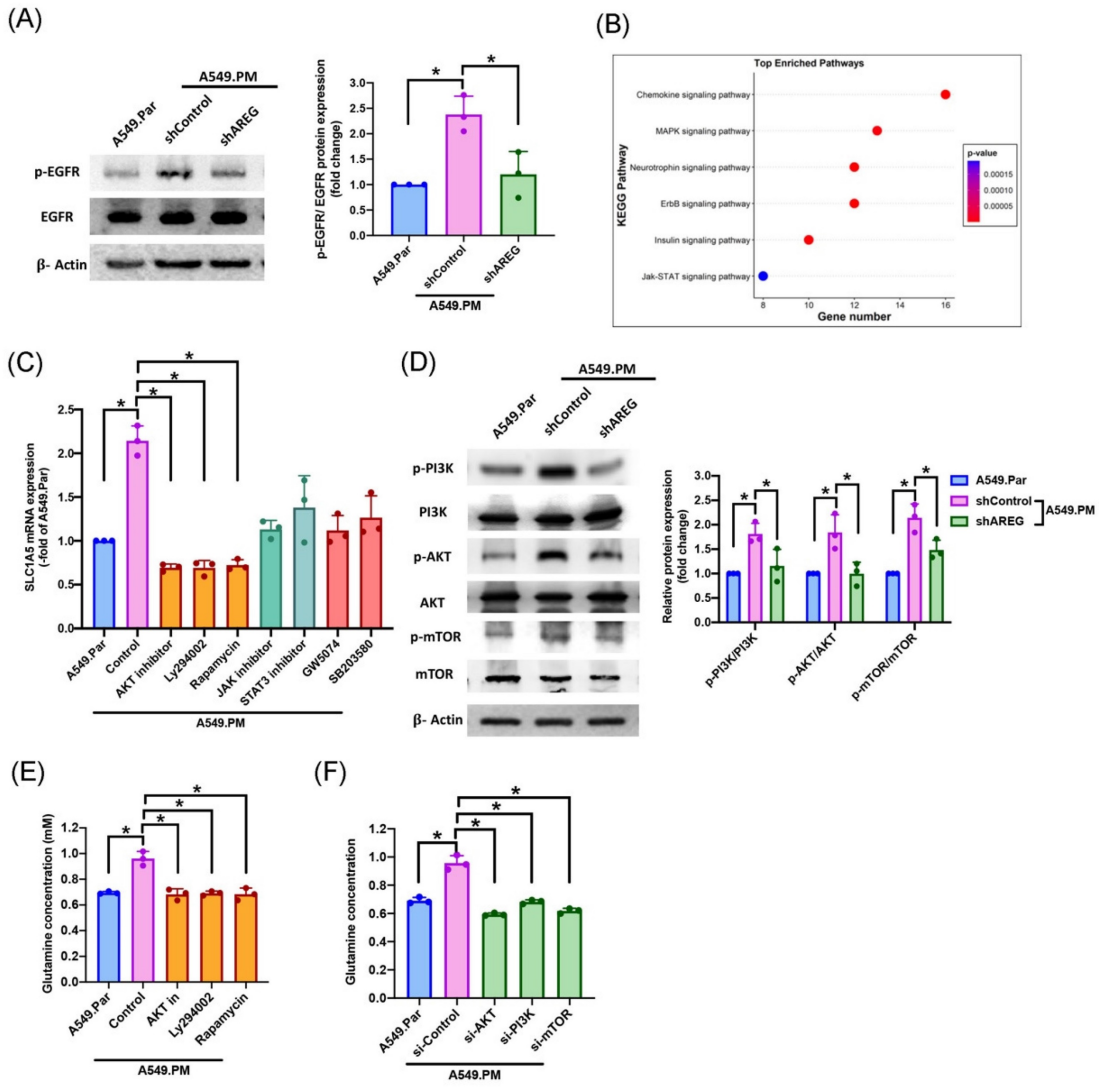
** PM-induced AREG expression promotes glutamine metabolism in lung cancer through the EGFR/PI3K/AKT/mTOR pathway.** (A) A549.PM cells were transfected with AREG shRNA for 24 hr. Western blot analysis examined levels of EGFR phosphorylation and β-actin. The graphs demonstrate densitometric analysis of p-EGFR/EGFR. (B) Signaling pathways in A549.PM cells were analyzed by RNA Sequencing. (C) Cells were treated with indicated inhibitors, and the expression of SLC1A5 was verified by qPCR. (D) A549.PM cells were transfected with AREG shRNA for 24 hr. Western blot analysis examined levels of PI3K, AKT, and mTOR phosphorylation and β-actin. The graphs demonstrate densitometric analysis of p-PI3K/PI3k, p-AKT/AKT and p-mTOR/mTOR. A549.PM cells were incubated with PI3K, AKT, and mTOR inhibitor or PI3K, AKT, and mTOR siRNA for 24 h. (E&F) Levels of glutamine expression were examined by glutamine Assay Kit. *p < 0.05 compared with A549.Par or A549.PM expression.

The AREG/EGFR axis induces tumor cell proliferation through multiple signaling pathways 11. We sought to determine the interaction between AREG and EGFR. Our results showed that when A549.PM cells were transfected with AREG shRNA, there was a significant decrease in p-EGFR level (Figure 7A). Next, we aimed to determine which signaling pathway is involved in PM promoting glutamine metabolism. RNA-Seq data also revealed that A549.PM cells significantly upregulated several EGFR downstream and proliferation signaling pathways such as chemokine, MAPK, neurotrophin, ErbB, insulin, and JAK/STAT signaling pathways (Figure 7B). RT-qPCR data demonstrated that when A549.PM cells were treated with PI3K (Ly294002) /AKT/mTOR (Rapamycin), JAK/STAT, and RAF (GW5074)/p38 (SB203580) inhibitors, the PI3K/AKT/mTOR cascade showed a greater decrease in SLC1A5 mRNA expression compared to the JAK/STAT and RAF/p38 cascades (Figure 7C). Our data also confirmed that transfection with AREG shRNA significantly decreased PI3K/AKT/mTOR phosphorylation expression (Figure 7D). Additionally, we showed that treatment of A549.PM cells with PI3K/AKT/mTOR inhibitors and their respective siRNAs inhibited glutamine concentration (Figure 7E&F). These results suggest that PM induces AREG generation through EGFR/PI3K/AKT/mTOR signaling, thereby increasing SLC1A5 to promote glutamine metabolism in lung cancer.

## 4. Discussion

Long-term exposure to PM is associated with an increased risk of lung cancer 36. Lung cancer cells stimulated with PM demonstrated higher proliferation, migration, and invasion of cancer cells 37. We found that long-term exposure to PM in lung cancer promotes the highest cell proliferation. Our *in vivo* data showed that A549.PM cells have higher tumor growth, tumor weight and tumor volume than A549.Par cells. This study elucidated the functions of PM-promoted lung cell proliferation both* in vitro* and *in vivo*.

The presence of AREG is closely linked to the oncogenic process; increasingly higher levels of AREG expression correlate with worse prognosis in several cancers, such as ovarian, glioma, head and neck, breast, and lung cancers 31, 38, 39. AREG overexpression has been demonstrated in a wide variety of human lung cancer tissues, and AREG is assumed to play an important role in promoting lung cancer proliferation 40. AREG also promotes tumor growth in pancreatic, colorectal, liver, and lung cancers 41-43. Our analysis of RNA sequencing data identified that long-term exposure to PM upregulates AREG expression in lung cancer cells. We also found that treatment with AREG shRNA reduces lung cancer tumor growth and cell proliferation compared to the A549.PM group. Our results suggest that targeting AREG could be a valuable therapeutic approach for lung cancer exposed with PM.

Metabolic reprogramming is a hallmark of cancer, and targeting metabolism is a potential therapeutic strategy 44, 45. Cancer cells mainly obtain nutrients through metabolic pathways involving glucose, fatty acids, glutamine, and small molecule amino acids to support their growth. During cancer cell growth and survival, glucose and glutamine are the main metabolic pathways that significantly influence it. Cancer cells break down glucose into pyruvate through glycolysis and utilize the high-energy compounds (ATP and NADH) and lactate released during this process to fuel their growth 34, 35. Additionally, cancer cells rely on glutamine-mediated TCA cycle to provide essential metabolites for growth 46. Our analysis of mass spectrometry data and seahorse assay identified that long-term exposure to PM increases glutamine concentration and OCR in lung cancer cells. Our findings also revealed that long-term exposure to PM increases glutamine metabolism, promoting lung cancer tumor growth and cell proliferation. As air pollution worsens, PM not only accumulates in the cytoplasm but also mitochondria and cell nuclei. This uptake activates cell signaling pathways, further promoting cell proliferation 47, 48. We also found that long-term exposure to PM increases the concentration of glutamine-derived TCA cycle metabolites such as glutamine, glutamate, alpha-KG, succinate, malate, citrate, and aspartate. PM augments AREG-dependent lung cancer proliferation through increasing glutamine metabolism. Therefore, regulating glutamine metabolism is a novel avenue for developing remedies for lung cancer growth exposed to PM.

SLC1A5 is responsible for transporting glutamine. Previous research has confirmed that SLC1A5 contributes to cancer progression by promoting glutamine metabolism 49. Many studies have shown that overexpression of SLC1A5 promotes progression in triple-negative basal-like breast cancer 49, prostate cancer 50 and lung cancer 51. Our study found that long-term exposure to PM increased SLC1A5 expression in lung cancer. We also found that high levels of AREG expression, promoted by PM, can upregulate SLC1A5 expression and enhance lung cancer tumor growth and cell proliferation. Our results suggest that the functional importance of glutamine transport mediated by SLC1A5 in cancer cell proliferation and survival, and SLC1A5 in the glutamine metabolism pathway, is an important therapeutic target for lung cancer.

AREG is a ligand of the epidermal growth factor receptor 9. Through binding to EGFR, AREG activates multiple downstream signaling pathways, including Ras/MAPK, PI3K/AKT, mTOR, and STAT 52, 53. These signaling cascades modulate gene expression and induce various cellular responses such as proliferation, survival, invasiveness, and angiogenesis 11. AREG, through PI3K/Akt signaling, induces its expression to promote epithelial ovarian cancer cell migration and proliferation 54. Our analysis of RNA sequencing data identified that long-term exposure to PM upregulates the PI3K/AKT/mTOR, JAK/STAT, and RAF/p38 signaling pathways. When A549.PM cells are treated with PI3K/AKT/mTOR, JAK/STAT, and RAF/p38 inhibitors, the PI3K/AKT/mTOR cascade significantly reduces SLC1A5 mRNA expression compared to the JAK/STAT and RAF/p38 cascades. We also found that AREG, via EGFR/PI3K/AKT/mTOR signaling, regulates glutamine concentration and SLC1A5 expression. These results suggest that the EGFR/PI3K/AKT/mTOR pathway plays critical role in PM-mediated glutamate metabolism in lung cancer. Our results illustrate that targeting AREG might offer a novel treatment approach for lung cancer. In the future, whether to use EGFR inhibitors such as erlotinib (Tarceva^®^) and gefitinib (Iressa^®^) for targeting AREG and decreasing long-term exposure to PM-induced lung cancer patients is worthy of further study.

Previous studies have demonstrated long-term exposure of mice to PM through intratracheal instillation 55, 56. A limitation of our research is that we didn't have the equipment for intratracheal instillation or the required expertise. Therefore, in our *in vivo* study, PM powder was utilized for long-term exposure to lung cancer cell lines and subsequently injected subcutaneously into mice. In future studies, using intratracheal instillation *in vivo* model, which more closely mimics natural exposure to PM, can be employed to more clearly explore how long-term exposure to PM increases the expression of AREG and promotes lung cancer growth through glutamine metabolism.

## 5. Conclusion

**Figure 8:**
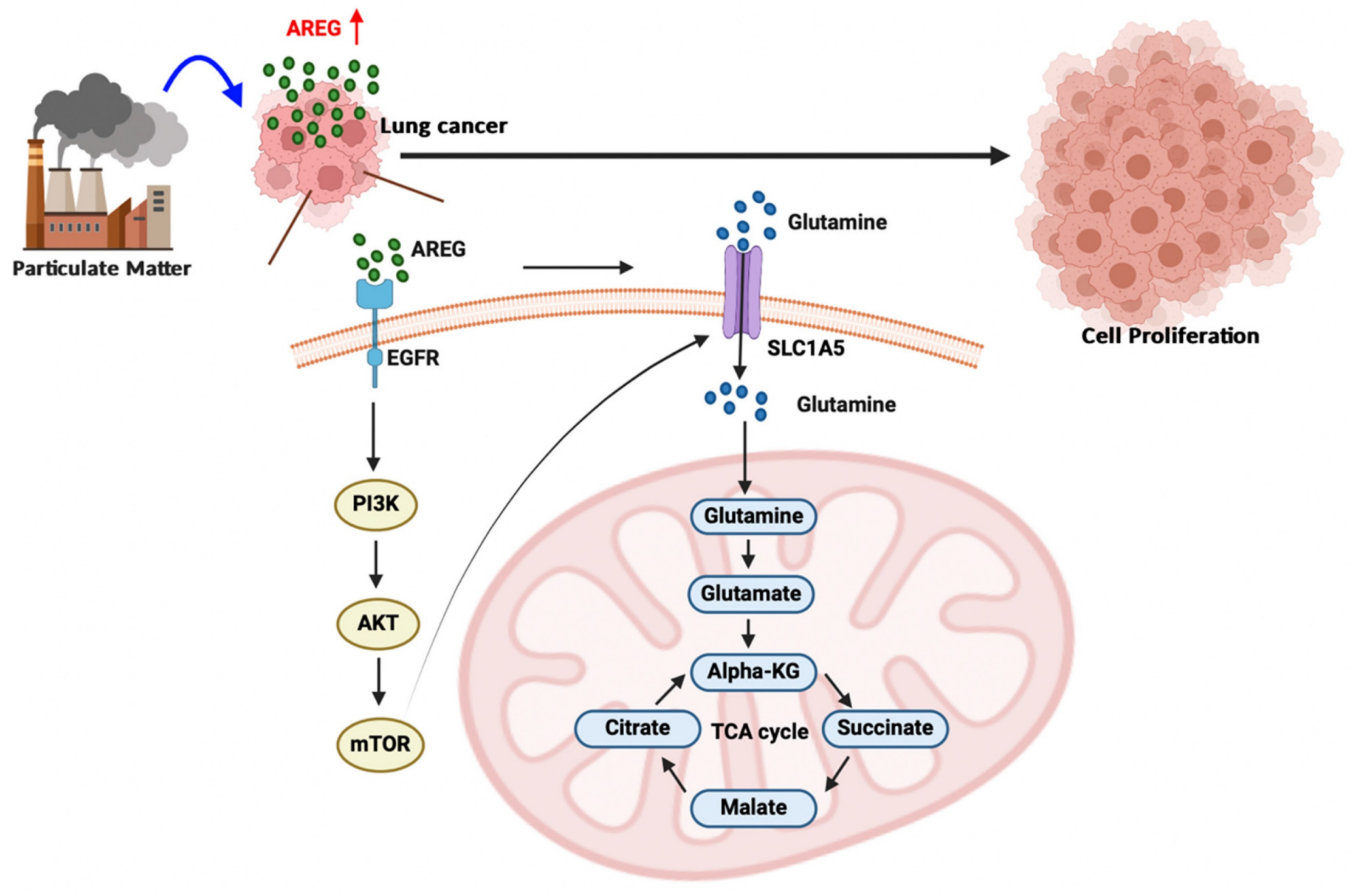
** A schematic diagram showing how PM increases AREG-dependent glutamine metabolism and lung cancer proliferation.** Long-term exposure to PM leads to increased AREG expression, which in turn induces SLC1A5 expression through the EGFR/PI3K/AKT/mTOR signaling pathway. The upregulation of SLC1A5 facilitates the accumulation of glutamine-derived TCA cycle metabolites, thereby promoting lung cancer cell growth and proliferation.

In conclusion, our study results demonstrate that long-term exposure to PM increases AREG expression in lung cancer cells. AREG, in turn, upregulates the glutamine transporter SLC1A5 and promotes the accumulation of glutamine-derived TCA cycle metabolites via the EGFR/PI3K/AKT/mTOR signaling pathway, thereby promoting lung cancer cell growth and proliferation (Figure 8).

## Supplementary Material

Supplementary materials and methods, table.
