# Supplementary material for: Particulate matter facilitates amphiregulin-dependent lung cancer proliferation through glutamine metabolism
Source: Int J Biol Sci. 2024 May 27;20(8):3126–39. doi: 10.7150/ijbs.96210 (PMC11186359; doi:10.7150/ijbs.96210)
Supplement: Supplementary file 1 — Supplementary materials and methods, table. [file ijbsv20p3126s1.pdf]

Particulate matter facilitates amphiregulin-dependent lung cancer proliferation through glutamine metabolism

Ya-Jing Jiang, Trung-Loc Ho, Chia-Chia Chao, Xiu-Yuan He, Po-Chun Chen, Fang-Ju Cheng, Wei-Chien Huang, Chang-Lun Huang, Po-I Liu, Chih-Hsin Tang

This file includes:

Supplementary Materials and Methods and Supplementary Table S1

Materials and Methods

Chemicals

The β-actin antibody, AKT (cat#A6730), mTOR (Rapamycin, cat#R0395), and JAK (cat#42099) inhibitors (cat#A5441) were purchased from Sigma-Aldrich (St. Louis, MO, USA). The AREG (cat#SC-74501), EGFR (cat#SC-53274), PI3K (cat#SC-1637), and AKT (cat#SC-5298) antibodies, STAT (cat#SC-204304), and RAF (cat#SC-200639) inhibitors were purchased from Santa Cruz Biotechnology (Santa Cruz, California). The SLC1A5 antibody (cat#NBP1-89327) was purchased from Novus Biologicals (Novus Biologicals, LLC). The p-EGFR (cat#2238s), p-PI3K (cat#4228s), p-AKT (cat#4060s), p-mTOR (cat#5536s), and mTOR (cat#2983s) antibodies were purchased from Cell Signaling (Danvers, MA, USA). The AREG and SLC1A5 short hairpin RNA (shRNA) were purchased from the National RNAi Core Facility Platform (Taipei, Taiwan). The PI3K (Ly294002, cat#ALX-270-038) and p38 (SB203580, cat#ALX-270-179) inhibitors were also purchased from Enzo Life Sciences (Farmingdale, NY, USA). The PI3K (L-003020-00-0005), AKT (L-003000-00-0005), and mTOR (L-003008-00) siRNA were purchased from Dharmacon (Lafayette, Colorado).

Cell culture

The human lung cell line A549 was purchased from the American Type Culture Collection (ATCC; Manassas, VA, USA). A549.Par cells were exposed to 25μg/mL of PM every two days for 60 days (long-term; A549.PM). The cell lines were cultured in Dulbecco's Modified Eagle Medium (DMEM) medium (Gibco, USA). Streptomycin (100μg/mL), penicillin (100 U/mL), N-(2-hydroxyethyl) piperazine-N'-(2-ethane sulfonic acid) (HEPES) (20 mM), glutamine (2 mM) and 10% fetal bovine serum (FBS;

Gibco, USA) was added to the cell culture medium. The cells were maintained at 37°C in a humidified atmosphere with 5% CO_2 [1].

Quantitative real-time polymerase chain reaction (RT-qPCR)

Total RNA was extracted from A549.Par and A549.PM cells using the TRIzol kit (Catalog No. 15596026) (MDBio, Taipei, Taiwan) and RNA concentrations were determined using the GE NanoVue Plus spectrophotometer (GE Healthcare Life Sciences, Pittsburgh, PA, USA). Complementary DNA (cDNA) was synthesized using the M-MLV Reverse Transcriptase kit (Catalog No. 28-025-013) (Invitrogen, Thermo Fisher Scientific, Waltham, MA, USA). The primers used in this study are listed in Supplementary Table S1. The qPCR method and the StepOnePlus™ sequence detection system for qPCR analysis were performed, following the protocol in our previous publications [2, 3].

Western blot

Total cell lysates were prepared from A549.Par and A549.PM cells. Cell lysates were prepared by RIPA buffer containing a protease inhibitor cocktail and the concentration was determined using the BCA Protein Assay Kit (Catalog No. 23225) (Thermo Fisher Scientific Inc., Waltham, MA, USA). Electrophoresis and transfer methods have been described in our previous study [4]. The membranes were blocked with 5% BSA at room temperature for 1 h, then incubated with primary antibodies (1:3000) for 1 h. After undergoing 3 washes in TBST buffer (0.05% Tween 20 in Tris-buffered saline), the blots were incubated with a secondary antibody. Band images were acquired using the ImageQuant™ LAS 4000 biomolecular imager (GE Healthcare Life Sciences, Pittsburgh, PA, USA) [5].

ELISA assay

Samples were obtained from A549.PM cells incubated with AREG shRNA. The samples were assayed using the AREG ELISA kit (ASIA BIOSCIENCE, Taipei, Taiwan), following the manufacturer's procedures.

Seahorse assay

The cells are seeding in the Seahorse XF96 plate. Then change the cell culture growth medium in the cell culture microplate to a warmed assay medium and place the cell culture microplate into a 37 °C non-CO_2 incubator for 45 minutes to 1 hour prior to the assay. Load oligomycin, FCCP, and Rot/AA into the ports on sensor cartridges. Then the mitochondrial respiration by Seahorse machine.

Immunohistochemistry (IHC) staining

Tumor cell sections were deparaffinized with xylene and rehydrated with ethanol. The NovoLink Polymer System (Catalog No. RE7150-CE) (Leica Microsystems) was used to perform IHC staining, according to the manufacturer's protocol in our previous study [6, 7]. Tumor tissues were acquired from the *in vivo* tumor progression model and then stained with human AREG or SLC1A5 antibodies, applied at a dilution of 1:100.

SUPPLEMENTARY TABLE

Supplementary Table S1. Primers sequences for RT-qPCR.

Genes	Forward (5'-3')	Reverse (5'-3')
PTGS1	CTCTGTGCCTAAAGATTGCCC	GTCTCCATAAATGTGGCCGAG
PTGES	TCCTAACCCTTTTGTCGCCTG	CGCTTCCCAGAGGATCTGC
PPAP2A	ACGCCCCACACTGCAATTT	TGAGTCCAGTCAACACATCGC
VAV3	CCAACCCTGGTATGCTGGAG	CCTGTGCCTCACAAGGTAAGT
BTC	CCTGGGTCTAGTGATCCTTCA	CTTTCCGCTTTGATTGTGTGG
AREG	GTGGTGCTGTCGCTCTTGATA	CCCCAGAAAATGGTTCACGCT
CDKN1A	CGATGGAACTTCGACTTTGTCA	GCACAAGGGTACAAGACAGTG
ALDH3A1	TGTTCTCCAGCAACGACAAGG	AGGGCAGAGAGTGCAAGGT
NOTCH1	TGGACCAGATTGGGGAGTTC	GCACACTCGTCTGTGTTGAC
ARRB1	CCTGACCTTTCGCAAGGACC	CAAGCCTTCCCCGTGTCTTC
TIAM1	ATGACGCTACATATTTGGCTGAG	ACCCAAGATTTCTTCGTTGCTT
CD38	CAACTCTGTCTTGGCGTCAGT	CCCATACACTTTGGCAGTCTACA
BST1	GTCCAGGCACTCTATTCCCAG	GACGGGTGTTGTCTGCAAAG
SLC1A5	TCATGTGGTACGCCCCTGT	GCGGGCAAAGAGTAAACCCA

References

1. Chang, E.M., et al., *PM(2.5) promotes pulmonary fibrosis by mitochondrial dysfunction.* Environ Toxicol, 2023. **38**(8): p. 1905-1913.
2. Chen, C.Y., et al., *CCN1 Promotes VEGF Production in Osteoblasts and Induces Endothelial Progenitor Cell Angiogenesis by Inhibiting miR-126 Expression in Rheumatoid Arthritis.* J Bone Miner Res, 2017. **32**(1): p. 34-45.
3. Chang, A.C., et al., *WISP-1 Promotes Epithelial-Mesenchymal Transition in Oral Squamous Cell Carcinoma Cells Via the miR-153-3p/Snail Axis.* Cancers (Basel), 2019. **11**(12).
4. Wang, M., et al., *Thrombospondin enhances RANKL-dependent osteoclastogenesis and facilitates lung cancer bone metastasis.* Biochem Pharmacol, 2019. **166**: p. 23-32.
5. Lin, C.C., et al., *Casticin inhibits human prostate cancer DU 145 cell migration and invasion via Ras/Akt/NF-kappaB signaling pathways.* J Food Biochem, 2019. **43**(7): p. e12902.
6. Liu, J.F., et al., *Thrombospondin 2 promotes tumor metastasis by inducing matrix metalloproteinase-13 production in lung cancer cells.* Biochem Pharmacol, 2018. **155**: p. 537-546.
7. Su, C.M., et al., *Resistin facilitates VEGF-C-associated lymphangiogenesis by inhibiting miR-186 in human chondrosarcoma cells.* Biochem Pharmacol, 2018. **154**: p. 234-242.
